# Removal of precursors and disinfection by-products (DBPs) by membrane filtration from water; a review

**DOI:** 10.1186/s40201-017-0285-z

**Published:** 2017-12-08

**Authors:** Mohammad Ali Zazouli, Laleh R. Kalankesh

**Affiliations:** 1 Department of Environmental Health Engineering, Health Sciences Research Center, Faculty of Health, Mazandaran University of Medical Sciences, Sari, Iran; 2 PhD student of Health Science, Student Research Committee, Department of Environmental Health Science, Health Sciences Research Center, School of Public Health, Mazandaran University of Medical Sciences, Sari, Iran

**Keywords:** DBPs, Drinking water, HAA5, Membrane technology, MF, NF, NOMs, RO, THMs, UF

## Abstract

Disinfection by-products (DBPs) have heterogeneous structures which are suspected carcinogens as a result of reactions between NOMs (Natural Organic Matter) and oxidants/disinfectants such as chlorine. Because of variability in DBPs characteristics, eliminate completely from drinking water by single technique is impossible. The current article reviews removal of the precursors and DBPs by different membrane filtration methods such as Microfiltration (MF), Ultrafiltration (UF), Nanofiltration (NF) and Reverse Osmosis (RO) techniques. Also, we provide an overview of existing and potentially Membrane filtration techniques, highlight their strengths and drawbacks. MF membranes are a suitable alternative to remove suspended solids and colloidal materials. However, NOMs fractions are effectively removed by negatively charged UF membrane. RO can remove both organic and inorganic DBPs and precursors simultaneously. NF can be used to remove compounds from macromolecular size to multivalent ions.

## Background

In recent years potable water security is considered as a worldwide issue. The need to remove pathogens from drinking water supplies is long recognized. Elimination microbial pollution from potable water through conventional water treatment methods is difficult. Disinfection of water refers to the inactivation or destruction of harmful organisms, especially pathogenic organisms of fecal origin, which living in the water [1, 2]. Among the different disinfection products, (DBPs) such as Halo acetic acids (HAAs) and Trihalomethanes (THMs) commonly show toxic effects on human health. Thus remove of them or its precursors are essential to avoid impact on public health [3–8]. Chlorine and chlorine compounds are common disinfectants which are added for disinfecting water at the most water treatment plants. During chlorination, chlorine can react with NOM and produce DBPs [9, 10]. In the last thirty years, because of potential health risks of DBPs in water, gained a lot of attention. According to several meta-analyses epidemiological, studies, chloroform are recognized carcinogen [11–13]. Therefore, the formation of it’s should be prevented. NOM [generally consists of Humic acid] are the most important precursors of DBPs. Chemical properties of NOM significantly effect on their removal efficiency [14]. NOM is a complex mixture of many chemical groups that varies both temporally and spatially [15, 16]. The NOM can be broadly divided into two fractions of hydrophilic such as alginic acid and hydrophobic fraction such as humic acid [17]. The major chemical groups in NOM are listed as humic species, carboxylic acids, amino acids, proteins and carbohydrates [18]. Hydrophobic NOM that contains hydrophobic acids (HPOA) can further be divided into humic acid, fulvic acids and (HPON). Carbohydrates, amino acids and carboxylic acids comprise much of the hydrophilic fraction (HPI), which is sometimes further split into hydrophilic acids (HPIA) and hydrophilic bases (HPIB) [18, 19]. Many different techniques have been used for removal NOM in water supplies. Since using of some conventional treatment processes such as coagulation, sedimentation and sand filtration are not completely efficient in the removal of organic matter [20]. Advanced treatment processes, including ozonation and activated carbon filtration are used after conventional treatment processes [21–23]. Ultrasono-oxidation, photo-oxidation processes [24] and degradation by nanoparticles [25] are used in water treatment processes. However, NOMs were responsible for DBPs formation and cannot remove easily by some processes such as coagulation [26–29]. Amy et al. reported that the majority of THMs precursors has a pore size less than 1 kDa. Therefore, membrane filtration (Ultrafiltration and Nanofiltration) has become an accepted method for removal of them. Over the years, several studies have been carried out and used membrane filtration to remove NOMs or its fractions from various water [30–34]. The removal efficiency of DBPs and NOMs with the filtration method was evaluated by some researchers. In additionally, employing ozonation followed by conventional treatment process can be useful to removal certain organic contaminants during drinking water treatment [35–37]. Recent studies demonstrate that removal of organic matter was significantly improved by hybrid process combining membrane [24]. The aim of this review article was reviewing different treatment processes for NOMs and DBPs removal in water treatment process with focus on membrane filtration. Also compare the advantage and disadvantage of each used method.

## NOMs as DBPs precursors

NOMs are various dissolved and particulate organic compounds which are generated with the decay of the plant, animal, and microbial tissue. Collectively, these organic compounds are known as Total Organic Carbon (TOC) in aquatic ecosystems. Dissolved partial of TOC "which is a subset of TOC" dissolves Organic Carbon (DOC) [38]. In the most of fresh water, nearly 83–98% of TOC is related to DOC [39]. TOC consists of organic compounds such as fats, waxes, terpenoids, tannins, lignins, cellulose, hemicelluloses, protein, sugars, and starches [40]. On the other hand, organic substance can be classified as humic and non humic compounds. Humic compounds constitute most of the natural organic matter in surface waters [41]. It was reported that the occurrence of DBPs in chlorinated water may vary significantly based on chlorine dose, bromide levels, and TOC. It has been demonstrated that the natural organic matter (NOM), especially the hydrophilic portion and amino acids, constitute important precursors for HAAs [42].

## Disinfection by-products (DBPs)

### Occurrence of DBPs

Since the beginning of the twentieth century, disinfection process has been routinely used to annihilate and inactivate pathogens in water. Chlorine and its’ compounds are a common alternative for disinfection of water [43]. Chlorine’s popularity is not only due to its’ lower cost, but it also produces large quantities of chlorine dioxide. The efficiency of the disinfection process depends on other conditions such as pH, temperature and contact time. Reactions between NOMs, with chemical treatment agents during disinfection process form DBPs. Typical DBPs include THMs, HAAs, and others, including iodine and fluoride. Generally, THMs and HAAs concentration are substantially higher than other organic DBPs classes. The first DBPs chemical class is Trihalomethanes (THMs) were discovered in 1974 [44].

### Toxicology of DBPs

In assessing the importance of disinfection in drinking water one shouldn’t neglect the toxicity associated with the disinfectant. United States National Institute of Cancer (NCI) is recognized that THMs are carcinogenic in the high dose, and raise the highest public health concerns [45]. Table 1 shows the possible health risks of DBPs and theirs guidelines and regulations which recommended by different organizations in the world. As well as, it shows that the most of them cause cancer, mutagenic and reproductive effects on human. There are relationships between DBPs in water and increasing the risk of some cancers such as bladder, stomach and colon cancers [46]. Some studies have reported adverse pregnancy outcomes including spontaneous abortion, low birth weight (LBW), small-for-gestational-age (SGA), stillbirth, and preterm delivery depending on DBPs [47].

**Table 1:** Toxicological effects, and DBPs (μg/L) guidelines and regulations [83-85]

Class of DBPs	Compounds	Health effects	CDWQ	USEPA	WHO	ISIRI
Trihalomethanes (THM)	Chloroform	Cancer, liver, kidney, and reproductive effects			0.2	
	Dibromochloromethane	Nervous system, liver, kidney, and reproductive effects			0.1	
	Bromodichloromethane	Cancer, liver, kidney, and reproductive effects			0.06	
	Bromoform	Cancer, liver, kidney, and reproductive effects			0.1	
Haloacetic Acid	Monochloroocetic Acid	Cancer and reproductive and developmental effects	80	60	a	0.200^b^
	Dichloroocetic Acid	Liver, kidney, spleen, and developmental effect	a		20	b
	Trichloroocetic Acid		0.050		50	
	Monobromoocetic		0.100		200	0.200
	Dibromoocetic Acid		a		a	
	Bromochloroacetic Acid		a		a	b
			a		a	
Haloacetonitrile (HAN)	Trichloroacetonitrile	Cancer, mutagenic and clastogenic effects				
Halogenated aldehydes and ketones	Formaldehyde	Mutagenic				
Halophenol	2-Chlorophenol	Cancer and tumor promoter				
Bromate	Bromide	Genotoxic carcinogen	10	10	10 (provisional)	b
	Bromate					
Chlorite	Chlorite	Irritation in the mouth, esophagus, or stomach, cancer or birth defects	100	1000	700(provisional)	b
	Chlorate					
Nitrosodimethylamine		Liver damage accompanied by internal bleeding, liver cancer and lung cancer, death of human babies	0.04 (proposed)	0.00069	0.01	b

*CDWQ* Canadian Drinking Water Quality, 2010, *USEPA* United States Environmental Protection Agency, 2012, *WHO* World Health Organization Guidelines, 2011, *IRISI* Institute of Standards & Industrial Research of Iran, 2009

^a^The sum of the ratios of the THM level to the WHO guideline values should not exceed

^b^Total index of THM (usually 70% of THMs compounds)

## Techniques for NOMs and DBPs removal

Several treatment processes can be significantly removed DBPs precursors. There are two methods for controlling DBPs in water. The first and most common strategy for controlling DBPs is removal of its precursors and use of alternative disinfectants such as enhanced coagulation, activated carbon adsorption, biologic treatment and nanofiltration [48–51]. The second, compliance, strategy is removing DBPs after formation which can prevent of the formation of THMs by several methods such as: membrane technology, air stripping and granular activated carbon [51–53]. Which technologies can prevent the formation of THMs are combination methods such as; ozone, monochloramines, hydrogen peroxide-ozone, UV-ozone and UV-hydrogen peroxide. The 99% of dissolved material and molecular weights in the 50 to 100 Da range can be removed by an RO membrane. Two important factors in successful rejection of contaminate are the membrane type and pore size [54].

### Membrane techniques

**Fig. 1:**
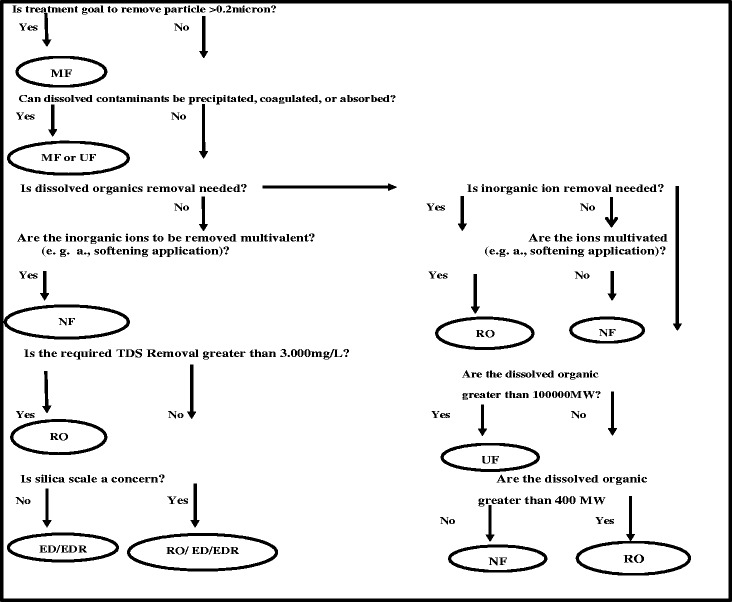
Generalized membrane selection chart [86]

Membrane technology was first observed in 1748 by Jean Antoine Nollet and it has used in water and wastewater treatment plants [55]. Also membrane techniques are proposed to remove THMs and their precursors from water. It also provides permeate quality far beyond the current regulatory requirement for potable water consumption [56]. The membrane is a selective barrier which separates particles and molecules by a sieving and diffusion mechanism [57]. Although the lowest concentration THMs are difficult to remove, water aeration and absorption of activated carbon have been traditionally used. However, aeration is not effective in the DBPs removal in comparing to adsorption on active carbon [58, 59]. RO, NF, MF, and UF are very similar technologies. Membranes are used in various applications. This is mainly due to their structure and preparation. Selecting a membrane to use depends on which contamination is present in the water. Figure 1 shows the choice of membrane filtration based on related questions to contaminant characteristics. The main problem of membrane of organic matter removal is fouling. Fouling reduces membrane efficiency and flux [17, 60, 61]. Therefore, water needs pretreatment before membrane processes.

#### Reverse osmosis (RO)

Reverse osmosis is pressure technology, which has been widely used for many purpose in water and wastewater treatment plants [54]. Nevertheless, the RO will not remove all contaminants from water, for example THMs, some pesticides, solvents, and other volatile organic chemicals (VOCs) are not effectively removed by reverse osmosis system. However, if the concentrations of the contaminations are not too high, RO systems can be a suitable alternative for removing VOCs, THMs, several pesticides and solvents [57]. As well as, some studies suggest that this technique has been the most effective water treatment technique for removal bromide and iodide. In addition, both organic and inorganic DBPs precursors can be removed by this technique simultaneously [62]. Also, Ro system should be used in the severely polluted water source or untreatable, a public water supply or a reliable private water source. Table 2 presents summary of some recent studies on Natural organic matter removal by Reverse osmosis membrane.

**Table 2:** Summary of some recent studies on removal NOMs and DBPs by RO membrane

Type of by product		Efficiency (%)	Type of membrane method	References
Precursors	Microorganism and Organic matter	89. 7	RO	[87]
		89.7	RO	[88]
	Humic Acid	100	RO/NF	[89]
		95	Polyamide forward Osmosis membrane	[90]
		98–99.3	RO	[33]
	NOM	99	RO	[91]
		44–90	RO	[92]
		97	Coupling RO/ Electro dialysis	[93]
	Dissolved Organic Matter (DOC)	90	RO/ Electro dialysis	[63]
		98.2	RO isolation	[94]
		90	RO	[95]
DBP_S_	THMs	83.8	MF/Active Carbon/RO	[96]
		80	RO	[97]
	Nitrosodimethylamine	66	RO / UV	[98]
		> 97	RO	[99]
	HAAs	60–90	RO	[100]
		83.77	RO / UV	[101]
	Bromide	>75	Electro dialysis Reversal	[102]
		70.48	RO / UV	[101]

#### Nanofiltration (NF)

Nanofiltration has been classified into pressure driven membrane process which represent an intermediate between Reverse Osmosis and ultrafiltration membrane processes, and exhibits features of both. Many types of membranes are used for drinking water treatment process, but the most applications of Nanofiltration are polyamide thin-film composite membranes in a spiral configuration. NOMs, small organic molecules and DBPs precursors can be effectively separated by NF membranes simultaneously [62]. NF has been recognized as a low pressure RO membrane. Patterson et al. reported that NF is a feasible process in the production of drinking water at small communities (populations of 25–500). This technique is able to reduce the pathogen and formation of potential DBPs precursors. In additionally, it could be a suitable alternative treatment, because of low-cost, easy operation and improve water quality to reduce consumer complaints [63]. Therefore, due to advantages of the technique, it can be widely applicator for water and wastewater treatment such as pharmaceuticals and personal care products (PPCPs) [60]. On the other hand, NF has the disadvantage of requiring extensive pre-treatment, high energy consumption brine disposal difficulties and especially fouling [61]. Again, like RO, this system is able simultaneously to remove both organic and inorganic DBPs precursors [64]. However, fouling of NF membrane system should be considered. Nevertheless, recent researches attempt to modify the surface of the membrane by chemical material such as grafting hydrophilic monomers, are not completely effective in reduction of membrane fouling [65]. Because the advantage of nanotechnology, applications for membrane technologies have expanded widely in water and wastewater treatment. Research communities reported that membrane fouling recently mitigated by nanoparticles based membranes [66, 67]. According to some reports, it is known that when Humic acids are added in membrane contained Nano particle, the HA molecules could be absorbed and filled the empty spaces between Nano particles which are on the surface of membrane [68, 69].

Table 3 illustrates a summary of studies on the use of nanofiltration to remove disinfection byproducts and their precursors.

**Table 3:** Summary of some recent studies on NOMs and DBPs removal by NF

Type of by product		Type of membrane method	Efficiency (%)	References
Precursors	Humic acid	NF	91–95	[32]
		Polyester NF	100	[103]
		Commercial NF/RO	100	[104]
	(NOM)	NF	58	[75]
		NF/RO/UF	93	[105]
		NF	49–100	[106]
	Dissolved Organic Matter(DOC)	UF/NF	70–99	[73]
		NF	*>*87	[107]
		UF/NF	98	[108]
		UF/NF	85	[109]
		NF	*>*90	[110]
		UF/NF	85	[75]
DBPs	THMs	NF	74–95	[111]
		NF	96–99	[112]
		NF**/** Air Stripping	42.97	[113]
DBPS	HAAs	NF	90–100	[114]
		NF	>95	[115]
		NF	80	[77]
	Nitrosodimethylamine	NF	57–83	[63]
		NF/RO	98	[116]

#### Ultrafiltration (UF)

Over the last 50 years, Ultrafiltration has been economically attractive as one of the most important technologies in various industrial water treatment processes. However, despite being cost effective, fouling is a limitation factor where increasing applied pressure drops and necessitates frequent cleaning. It takes place due to microbial growth colloidal and scale precipitation [70]. To prevent fouling, a variety of pretreatment alternatives have been investigated to remove NOMs from water, such as coagulation, active carbon absorption, absorption of iron oxides other preformed settle able solid phases, or ozonation [71, 72]. UF is recognized to reduce turbidity, suspend solids and particles, but this method isn’t effective in separating humic substances which have high THMs and HAAs formation potential, however, NF can effectively remove THMs precursors [71]. UF membranes are known with different membrane materials and wide pore size range distribution as well as different surface charge densities. And it doesn’t directly predictable removal NOMs by size exclusion. Charged UF membranes have shown much higher removals of NOMs compound, whereas lowest removals can be obtained with uncharged membranes [73, 74]. On the other hand, NOMs compounds are too small to be retained by the pores of ultrafiltration membranes effectively [75, 76]. According Table 4, some studies reported that removal of DBPs precursors in lab scale tests be quite effective by UF membranes while assailable organic carbon (AOC), cannot be removed successfully by this treatment method. AOCs are compounds which have MM *<*1 kDa and responded to 30–40% of TOC in water. Acetate, amino acids and carboxylic acids are classified in AOC compounds [77]. Because of linear configuration and a larger radius structure, NOMs can be removed easily by both the size exclusion and charge repulsion in higher pH levels. Charged membranes are affected by pH much greater than neutral membranes. UF is as a suitable alternative pretreatment step for NF and RO which is able to remove microorganisms.

**Table 4:** Summary of some recent studies on NOMs and DBPs removal by UF membrane

Solution	Membrane, material,cut-off*/*pore diameter, module type, TMP	Quality of permeate: content and*/*or removal %	Variable	Removal%	Reference
Humic acid (Aldrich) 2 mg L^−1^, DOC 8.7 mg L^−1^, different pHs	Different flat sheet membranes or charges, stirred cell, lab scale,69 kPa	55	RC, 100 kDa, neutral, pH 3.5		[117]
		59–97	RC, 100 kDa, neutral, pH 7.5		
		79	RC, 100 kDa, charged, pH 3.5		
		92–98	RC, 100 kDa, charged, pH 7.5		
		66	PES, 100 kDa, zeta −12.3 mV		
Surface water, TOC 2.3 mg L − 1, SUVA 1.7, THMFP 70 μgL^−1^	Different flat sheet membranes, stirred cell, lab scale	25	NTR-7410, S-PSu, 20 kDa	20	[118]
		47	GR90, PSu, 10 kDa		
		50	ETNA01A, HPC, 1 kDa	44	
		53	HEKLA01A, amine + DIC, 1 kDa	48	
Different surface and groundwater	GM, PA TFC, 8 kDa, flat sheet, tangential cross-flow, bench scale	64	DOC 2.0 mg L − 1, SUVA 2.4	38	[119]
		84	DOC 3.9 mg L − 1, SUVA 4.4	60	
		93	DOC 9.8 mg L − 1, SUVA 4.9	85	
		93	DOC 6.8 mg L − 1, SUVA 5.7	87	
Reservoir water, DOC 4.0 mg L − 1, SUVA 2.0	Ceramic, 4 nm, single channel tubular, lab	UV28072	TMP 400 kPa	55	[120]
		UV280 83	TMP 1200 kPa	75	
Humic acid (Aldrich), DOC 10 mg L^−1^	KERMBMU1, Ceramic, 15 kDa*/*3.54 nm, single tubular, bench scale	85	pH 2.4, zeta −2.9 mV, pI 1 mmol	59	[119]
			pH 7.9, zeta −15.6 mV, pI 1 mmol	99	
Natural water, DOC 3.4 mg L − 1, SUVA 2.5, HMM ∼ 12 kDa, LMM ∼ 1.8 kDa	Different flat sheet membranes, cross-flow, lab-scale	HMM	PT, PES, 5 kDa	61	[75]
		LMM 7,			
		THMs 60 μg L − 1 63,			
		HAAs 34 μg L − 1 38,			
Moorland water, TOC 9.8 mg L^−1^	PSu, 100 kDa, flat sheet, bench scale, 100 kPa	22		18	[108]

#### Microfiltration (MF)

Microfiltration is a kind of physical filtration process which is commonly accepted for a removal p article matter of water. This technology can be used for both as a pretreatment step or as a water treatment process alone. On the other hand, this process is utilized for waters with high turbidity as a treatment process or as a pretreatment process for NF or RO [78, 79]. Dissolved organic carbon cannot be easily removed by MF, unless they are associated with particulates [80]. MF membranes are produced with various materials such as polymers, ceramics and metals, but only Polymeric and ceramic membranes are used in the water treatment field. Low cost, easy to scale up, and easy variation in module form is the most important advantages of polymeric membranes compared to ceramic membranes. So, they are commonly applied in water treatment process [81]. Ceramic membranes have longer life span, excellent chemical resistance, thermal stability and they are thermally generable from used membranes, so they are widely used in chemical processing [82]. This kind of membrane has been investigated for NOMs removal and it is clearly observed that MF membranes have a pore size much larger than the NOMs particles. Hence, they are ineffective for NOMs removal, beside their tendency to stick to the pores and deposit onto the membrane surface, which eventually, causes pore blocking. As a result, membrane fouling can be controlled by coagulation/flocculation as pretreatment.

## Conclusion

Natural Organic Matter (NOMs) and biopolymers and their degradation product in surface water can react with disinfection and cause different problems in drinking water treatment and water supply systems and health risk. Differences in NOMs Composition make it difficult to remove completely. The most important properties of membrane filtration are pore size. By decreasing of membrane pore size, removal of NOMs is increasing.

Although, MF membranes have large pore size, but unable remove NOMs, unless be obtained bigger flocks by coagulated. This membrane is a suitable alternative to removal suspended solids and colloidal materials such as, pathogens and algae, as well as economically acceptable than tighter membranes. Larger hydrophobic NOMs fractions are removed effectively by negatively charged UF membrane, even on the basis membrane would be expected more cutoff value and NOMs size. These kinds of membranes have some advantages. First, NOMs with small hydrophilic compounds and acidic content are removed difficultly by size or charge exclusion by UF membranes. Secondly, AOC and bacterial re-growth potential in drinking water distribution systems be controlled by UF membrane efficiently. For obtaining the required water quality, MF and UF with combined by another process such as adsorption, coagulation, oxidation */*BAC or tighter membranes (NF or RO) should be applied at the same time.

Nanofiltration can be used to remove compounds from macromolecular size to multivalent ions. By the sensitivity of NF membrane to fouling, Extensive pretreatment (include MF*/*UF, conventional treatment or slow sand or dual media filtration) are required to control colloidal, organic and biological fouling and scaling.

RO is cost effective membranes which can remove 99% of dissolved material. This technique can remove both organic and inorganic DBPs precursors simultaneously, and make it invaluable in DBPs minimization. However, RO remains relatively expensive, requires extensive pretreatment, has high energy consumption due to high operating pressures, and is susceptible to scaling, as well as brine disposal difficulties. The capital and operational expenses of RO, as well as the disposal of the generated concentrate are currently restricting of the widespread application this technique in drinking water treatment plants.
